# Mast cell glycosaminoglycans

**DOI:** 10.1007/s10719-016-9749-0

**Published:** 2016-11-30

**Authors:** B. Mulloy, R. Lever, C. P. Page

**Affiliations:** 1 Sackler Institute of Pulmonary Pharmacology, Institute for Pharmaceutical Science, King’s College London, Franklin-Wilkins Building, 150 Stamford St, London, SE1 9NN UK; 2 1 UCL School of Pharmacy, Brunswick Square, London, WC1N 1AX UK

**Keywords:** Mast cell, Heparin, Chondroitin, Dermatan, Glycosaminoglycan, Serglycin

## Abstract

Mast cells contain granules packed with a mixture of proteins that are released on degranulation. The proteoglycan serglycin carries an array of glycosaminoglycan (GAG) side chains, sometimes heparin, sometimes chondroitin or dermatan sulphate. Tight packing of granule proteins is dependent on the presence of serglycin carrying these GAGs. The GAGs of mast cells were most intensively studied in the 1970s and 1980s, and though something is known about the fine structure of chondroitin sulphate and dermatan sulphate in mast cells, little is understood about the composition of the heparin/heparan sulphate chains. Recent emphasis on the analysis of mast cell heparin from different species and tissues, arising from the use of this GAG in medicine, lead to the question of whether variations within heparin structures between mast cell populations are as significant as variations in the mix of chondroitins and heparins.

## Introduction

**Fig. 1:**
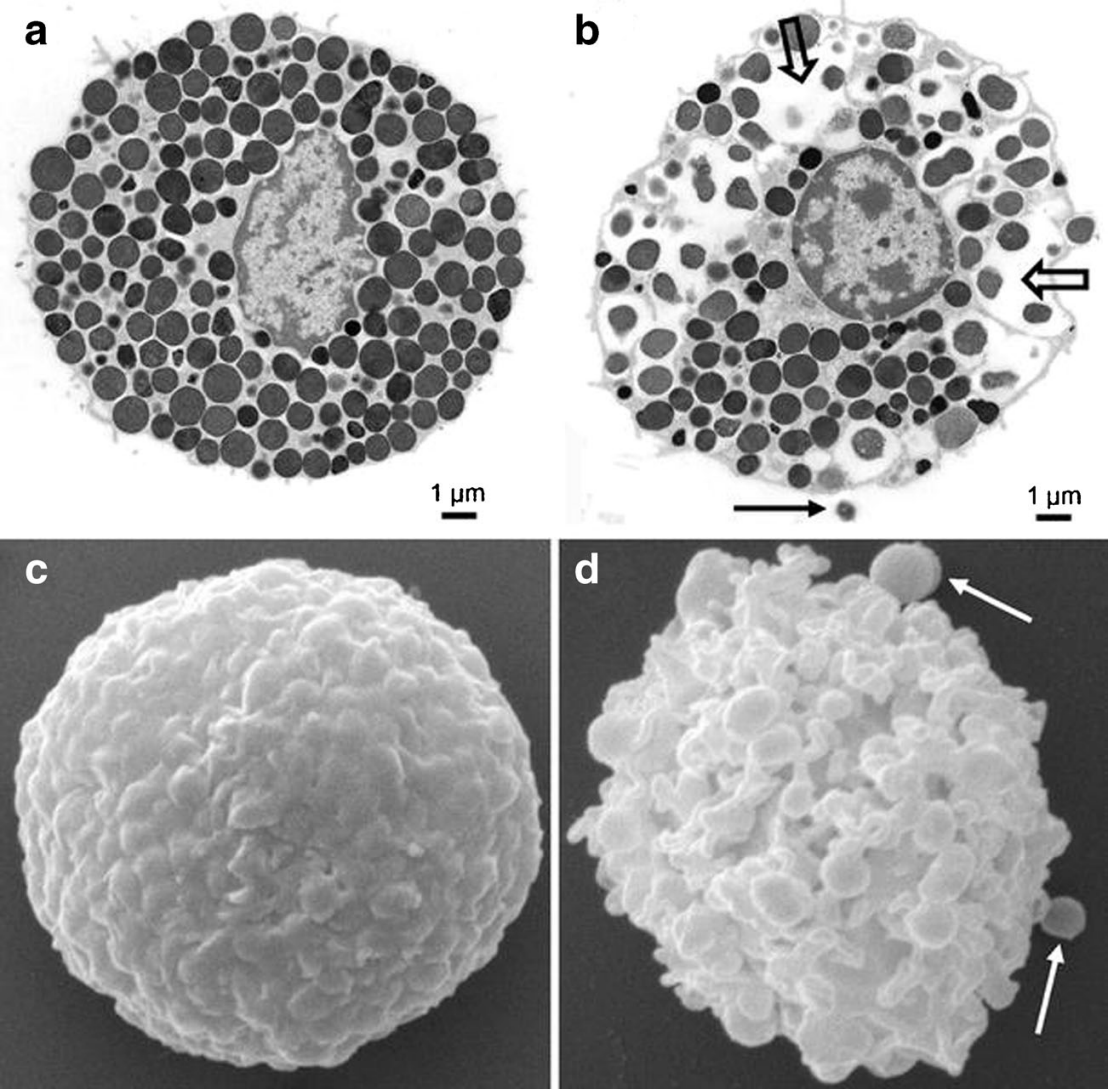
**a, b** Transmission electron micrographs showing an intact rat peritoneal MC (a) and a MC undergoing anaphylactic degranulation (**b**). The block arrows indicate regions where multiple granules have fused; the arrow depicts an exocytosed granule remnant. c, d Scanning electron micrographs showing an intact (**c**) and degranulated (**d**) rat peritoneal MC. Note the extensive membrane alterations in d. Arrows in d depict exocytosed granule remnants. Images are courtesy of Prof. Giuliano Zabucchi, Prof. Maria Rosa Soranzo, and Dr. Francesca Vita (Electron Microscopy Section of Centro Coordinamento e Sviluppo Progetti e Apparecchiature (CSPA), University of Trieste). Figure and caption reproduced with permission from [39]

Mast cells originate from bone-marrow derived leukocytes, and are distributed to tissue locations outside the vasculature before maturing under the influence of stem cell factor (SCF) and IL-3 to phenotypes characteristic of each tissue [1–3]. The most striking feature of mast cells is their large secretory granules (Fig. 1), that contain a formidable arsenal of proteases, small molecule biogenic amines, and cytokines ready for release when mast cells degranulate as a response to external stimuli, in a rapid pro-inflammatory reaction to the possible presence of an invading organism.

The granule contents are closely packed, with the help of the proteoglycan serglycin, and its GAG sidechains [4]. The intracellular GAGs stored in granules of mast cells (and a few other cell types, such as basophils) are an interesting set of compounds. GAGs are largely thought of as being displayed to the extracellular space, on the surfaces of cells and in the extracellular matrix. In this context they may play structural roles, or may interact with morphogens, cytokines and other small protein messengers, guiding their location in tissue or acting as cell surface receptor components [5]. The structures and roles of mast cell granule GAGs are not at present a well-pursued research topic, with the exception of studies concerning the HS variant known as heparin. This polysaccharide is isolated from intestinal mucosa of pigs, cattle and sometimes sheep in kilogram quantities, to be used as an antithrombotic agent in medicine [6]. Given its location in mast cell granules, it is not clear what the native function might be of the potent anticoagulant activity of heparin, mediated by its high-affinity, specific interaction with the plasma serine protease inhibitor (serpin) antithrombin [7]. We believe that the time is ripe for a revival of interest in the structures and functions of mast cell granule GAGs.

## Serglycin, the mast cell granule proteoglycan

The mast cell granule proteoglycan, serglycin, has a small protein core; the human sequence is 158 amino acids long, including an N-terminal signal peptide. It contains a central domain in which serine and glycine alternate; the human sequence has 8 serines in this sequence, and the mouse has ten, whereas the rat sequence is much longer, containing 24 serines [8]. Serine residues in this region may bear galactosaminoglycan sidechains, either chondroitin-4-sulphate (CS-A) or dermatan sulphate (DS; CS-B); or glucosaminoglycan chains of the heparan sulphate/heparin family. It has not been established whether the same protein core carries both types of GAG chain or whether they are segregated in different proteoglycan molecules.

**Fig. 2:**
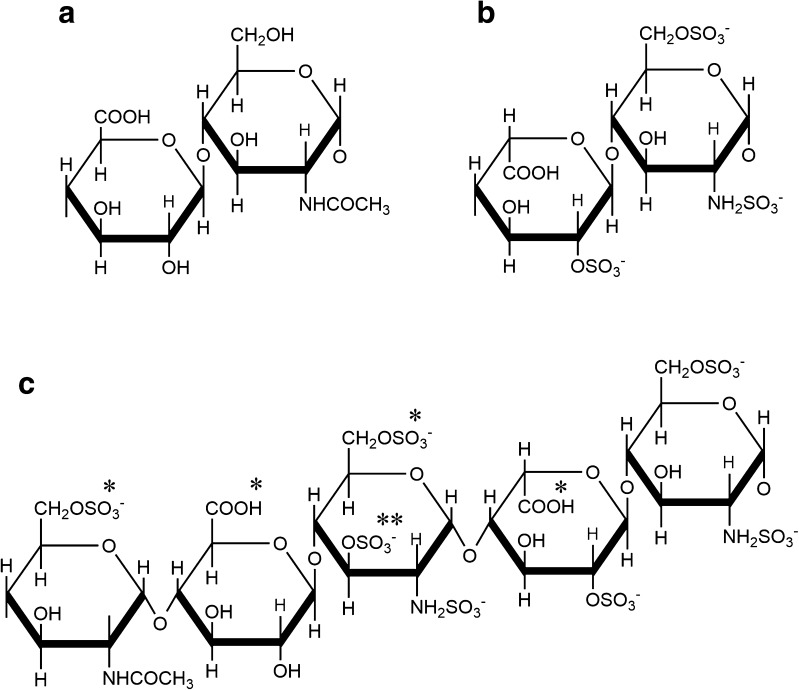
GAG structures found in mast cells. **a** The main repeating unit of heparan sulphate, [ −4)-β-D-GlcA-(1 → 4)-α-D-GlcNAc-(1- ] (GlcA-GlcNAc). This structure forms the precursor polysaccharide, which is transformed by a series of enzymes into **b** the main repeating unit of heparin [−4)-α-L-IdoA(2SO_3_^−^)-(1 → 4)-α-D-GlcNSO_3_^−^(6SO_3_^−^)-(1-] (IdoA2S-GlcNS6S). **c** the pentasaccharide with high affinity for antithrombin, as found in porcine mucosal heparin; essential substituents are marked with an asterisk. The enzyme 3-*O*-sulfotransferase (3-OST) isoform 1 adds the unusual 3-*O*-sulphate substituent (indicated with a double asterisk) to the central *N*-, 6-disulphated glucosamine in the GlNR6S-GlcA-GlcNS,6S–IdoA2S-GlcNS,6S sequence

**Fig. 3:**
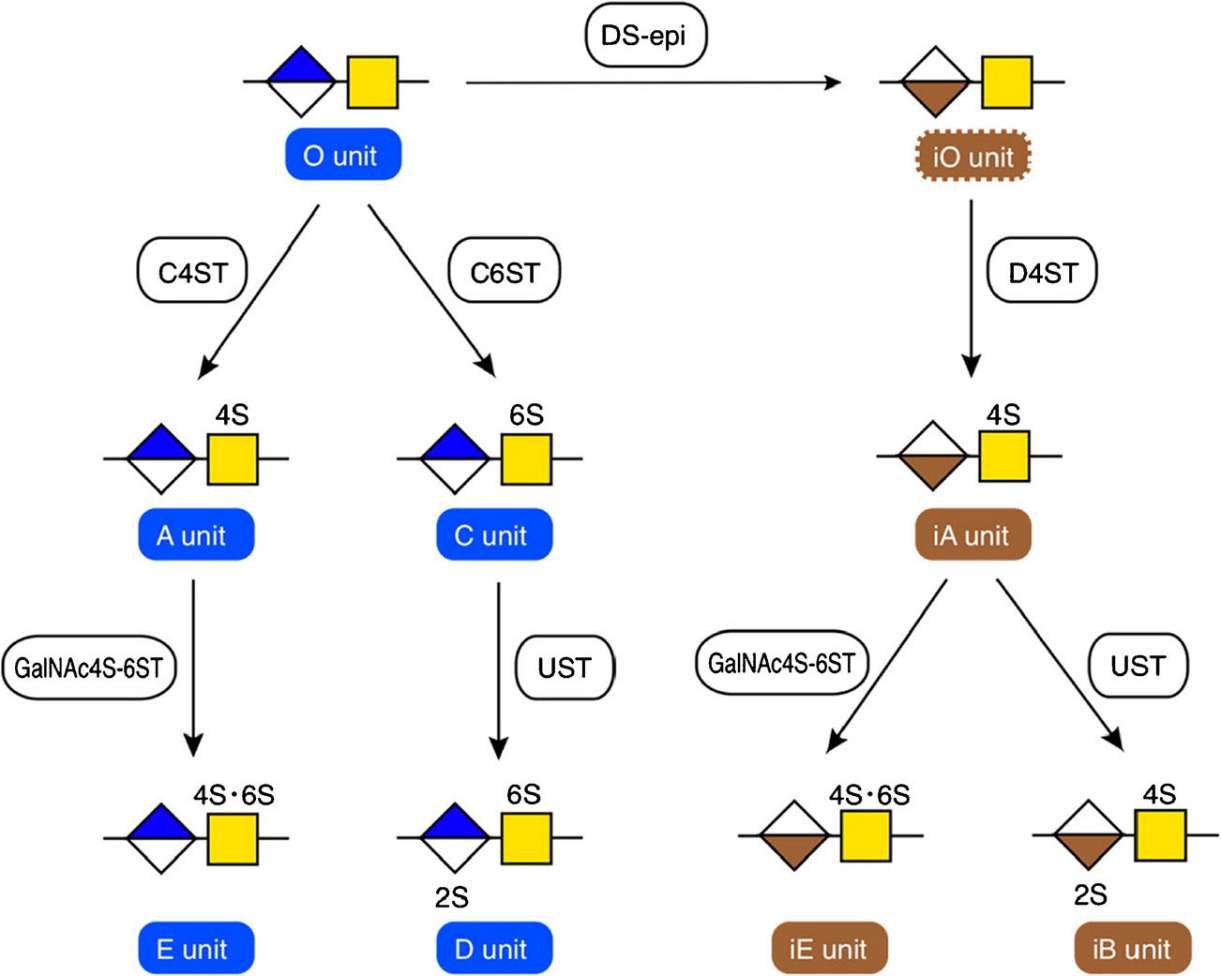
The conversion of the chondroitin backbone to CS-A, CS-B, CS-C, CS-D, CS-E and CS-diB by the enzymes chondroitin-4-sulfotranferase (C4ST), GlcA 5-epimerase (DS-epi), dermatan 4-*O*-sulfotransferase (D4ST), chondroitin 6-sulfotransferase (CS6ST) uronosyl 2-*O*-sulfotransferase (UST) GalNAc 4-*O*- sulphate 6-*O*-sulfotransferase (GalNAc4S-6ST). Reproduced with permission from [15]

In 1971, a macromolecular form of heparin was prepared from rat skin, with a molecular weight of more than 100 kDa [9], and later the complete rat peritoneal mast cell heparin proteoglycan, serglycin, was found to have a molecular weight of about 750 kDa, with heparin chains of about 75 kDa attached to a protease resistant core [10]. The term ‘serglycin’ came into use later in that decade [11, 12]. Serglycin varies in its GAG substitution between different cell types and between sub-populations of mast cells in different environments [13, 14], as discussed below. The GAG substituents of serglycin make up most of its molecular weight, and variability in the number and length of polysaccharide sidechains, together with the difficulty of isolation of whole serglycin, probably explains why the estimated molecular weight of serglycin varies between 60 KDa and 750 kDa [4]. Estimates at the higher end of this range lead to the conclusion that most of the serines in the alternating SG sequence are GAG substituted, implying a tightly packed, conformationally restricted environment for the GAG linkage sequence and nearby GAG repeating sequence (Figs 2 and 3).

## Heparin and chondroitin sulphate in mast cell granules: biosynthesis of mast cell GAGs

The GAG biosynthetic machinery of mast cells is similar to that of any other cell. The linkage sequence, −3Galβ1–3-Galβ1–4-Xyl-O-Ser, is common to both the galactosamine and glucosamine containing types of GAG. The next monosaccharide is glucuronic acid (GlcA), added by the enzyme GlcAT-1, and after that either GalNAcT-1 or GlcNAcT-1 commits the site to either chondroitin or heparin/HS substitution, respectively [5]. GAGs are built up from this common linker sequence of sugars by a series of enzymes that elongate the linear polysaccharide chain, add sulphate substituents, and epimerise some GlcA residues to α-L-iduronic acid (IdoA); the processes for biosynthesis of the chondroitins [15] and for heparan sulphate [16] (Figs 2a and b) have been described. There is, however, a difference between the biosynthetic pathways for HS in other cell types and mast cell heparin, in the first of the post-polymerization enzymes to modify the polysaccharide chain; *N*-deactylase, *N*-sulphotransferase (NDST). The isoform NDST-1 introduces short sequences of *N*-sulphated residues into the HS structure; the isoform NDST-2 introduces much longer, almost continuous *N*-sulphated domains into heparin [17].

Mast cell GAGs are highly sulphated, and there are several sulphate-related structural motifs associated with the particular functions of mast cell heparin and chondroitin sulphates. Best understood, perhaps, is the pentasaccharide structure in heparin that has high affinity for antithrombin (Fig. 2c). Though this is not a common sequence, it is more abundant in heparin than in most heparan sulphates [18]. The central glucosamine in the pentasaccharide bears three sulphates; it is 3-*O*-sulphated, besides having regular 2-*N*-sulphation and 6-*O*-sulphation. The 3-*O*-sulfotransferase (3-OST) that introduces this substitution has several isoforms, and it is 3-OST-1 that is effective in the mast cell [19]. The glucosamine substrate residue is preceded (at the nonreducing side) by GlcA rather than IdoA.

The presence of disulphated disaccharide units in chondroitin sulphate from mast cells has long been recognised. Two of these structural units are commonly encountered, the 4,6 sulphated CS-E motif and the 2,4 sulphated CS di-B motif (Fig. 3). The enzyme responsible for the CS-E motif, *N*-acetylgalactosamine-4-sulphate-6-*O*-sulphotransferase, was shown to be expressed in mouse mast cells, and to act on internal as well as non-reducing terminal GalNAc4S [20]. The CS di-B disaccharide is achieved by sulphation of IdoA in the 4-*O*-sulphated dermatan repeat disaccharide to IdoA2S, by the uronosyl-2-*O*-sulphatase (UST) enzyme, also responsible for converting 6-sulphated CS-C to CS-D (Fig. 3) [21]. The occurrence of several di-B disaccharides in succession gives rise to the high-affinity sequence for heparin cofactor II, a thrombin inhibiting serpin that can be activated by either heparin or DS [22].

### GAGs in mast cell sub-populations and in other leukocytes

Research in rodent species led to the definition of two mast cell subtypes, the connective tissue mast cells (CTMCs) and mucosal mast cells (MMCs); CTMCs are constitutive and T cell independent whereas MMCs are induced and T-cell dependent [23]. The maturation process of the mast cells also results in differences in granule contents, for example different profiles of granule proteases. They are also histochemically different; CTMC granules can be stained with safranin, whereas MMCs cannot, though both types of cell can be stained with alcian blue. This has been attributed to systematic differences in GAG content of the two cell types. The fluorescent dye berberine has also been used to stain CTMC granules, but does not stain MMCs from the intestine [24]. Berberine and safranin staining were used to demonstrate that cultured mast cells could take on the characteristics of CTMCs or MMCs when transplanted into mast cell deficient mice, depending on the tissue to which they were introduced [25], and in fact both CTMCs and MMCs originate from the same precursor bone marrow mast cells [23].

The statement sometimes encountered, that heparin is only found in connective tissue mast cells, and not in mucosal mast cells, is a generalisation based on studies on rodent mast cells in the 1970s and 1980s. Certainly, heparin has been identified in CTMCs from rat peritoneum [26] and found to be present in minor amounts, or completely absent, in MMCs from the intestines of nematode-infected rats [27–29]. In humans, both connective tissue mast cells and mucosal mast cells were found to contain GAGs with high affinity for antithrombin, using an antithrombin-gold stain [30]. The same system was able to distinguish between rat CTMCs and MMCs. It may be that humans do not have the exact equivalent of rodent MMCs.

The GAGs of rat leukaemic basophils have been identified as chondroitins 4- and 6-*O*- sulphate, dermatan sulphate, and heparin [31]. Using disaccharide analysis by HPLC against standard compounds, GAGs from platelets and mixed granulocytes were found to be of the CSA/DS type, with a small amount of CS-E and CS-diB.

Recently, detailed analysis of leukocyte GAGs has identified 4-*O*-sulphated CSA (not DS) as the major GAG component in several cell types, with a small proportion of a highly sulphated heparin-like HS (the study did not separate granule from cell surface GAGs, but assumes that the proportion of cell surface GAGs is small) [32].

## Fine structure of mast cell GAGs

The most common method for analysis of mast cell GAGs is enzymatic depolymerisation followed by chromatographic or electrophoretic separation and quantitation of the resulting oligosaccharides. The availability of chondroitinase enzymes, and the ability to separate the oligosaccharide products of chondroitinase digestion, meant that more detailed analysis of the chondroitins found in mast cells was possible in early studies than was the case for heparin/HS-like GAGs. The chondroitin sulphates of rat MMCs have been described as a mixture of CSA and CSE [27], or as a mixture of CSA and CSdi-B [28] or as a mixture of monosulphated CS, with both CSE and CSdiB [29]. In all cases, the disulphated CS elements, such as CS-diB and CS-E, are found together with monosulphated, particularly CS-A disaccharides.

It has been pointed out that the use of chondroitinase digestion leaves the nature of the original uronic acid in each disaccharide uncertain, as IdoA and GlcA give rise to the same unsaturated uronic acid at the non-reducing end of cleaved oligosaccharides; so, for example, the 2,4-*O*-disulphated disaccharide (CS-diB), though it is generally assumed to come from dermatan sulphate, might conceivably originate from a chondroitin polysaccharide containing a motif in which the GlcA is 2-O-sulphated [29]. Certainly, the ‘CS-A’ disaccharides could as easily be derived from dermatan sulphate as from CS-A itself, and indeed this has been shown to be the case for rat peritoneal mast cells, in which the 4-*O*-sulphated galactosaminoglycan is susceptible to chondroitinase ABC but not to chondroitinase AC, which does not cleave dermatan (CS-B) [33]. This has recently been confirmed by direct NMR spectroscopic analysis of rat peritoneal mast cell GAGs [35].

More recently, it has become possible to perform disaccharide analysis on heparin/HS as well as on CS, as for example in a study of bone marrow derived mast cells of Ctr2^−/−^ mice (Ctr2 is a protein that regulates mobilisation of endosomal Cu pools). The Ctr2^−/−^ mast cells contained more heparin and less CS than wild type controls [34], with a considerable increase in 6-*O*-sulphation of glucosamine due to increased expression of the HS6ST-1 sulfotransferase enzyme. A particularly interesting study of GAGs derived from leukocytes of individual human subjects was able to compare disaccharide composition and CS chain lengths for polymorphonuclear cells (PMNs), peripheral blood mononuclear cells (PBMCs), T- and B-cells, natural killer (Nk) cells and monocytes [32], identifying a remarkable consistency in the disaccharide composition of leukocyte HS/heparin between subjects.

As the sequence of serglycin does not vary between mast cell subtypes in a single species, differences in their proportions of heparin/HS and chondroitin GAG substituents cannot be dependent on the peptide sequence near the linkage site. It may be that the mechanism through which the local mast cell environment can determine the GAG type on its serglycin is related to the GalNAc-T1/GlcNAc-T1 stage of GAG chain initiation; in addition other influences such as availability of nucleotide monosaccharide donors and the sulphate donor 3′-phosphoadenosine-5′-phosphosulphate (PAPS) may play a part.

## Interactions between GAGs and other granule contents

The many small molecules and peptides found in mast cell granules have recently been listed [2]. They include small molecules such as biogenic amines, and a set of mast cell specific proteases as well as many other proteins. These proteases differ between species, in particular between human and rodent mast cells.

The storage of histamine and serotonin in mast cell granules is dependent on the presence of serglycin [36]. Histamine can bind to heparin at a density of one molecule of histamine to one disaccharide unit in a highly ordered fashion [37]. The relatively low pH of mast cell granules increases the affinity of this electrostatic interaction, and the increase in pH on release into surrounding tissue reduces affinity.

## GAGs and mast cell proteases

The proteases specific to mast cell granules are chymases, tryptases and carboxypeptidase A3; in human mast cells there is one chymase and a limited repertoire of tryptases compared with the numerous mouse enzymes [2]. In addition to these, several other proteases occur in mast cell granules but are not specific to them; examples are cathepsin G, granzyme B [2], and cathepsin E, which cleaves the carboxypeptidase A precursor to give the active enzyme [38]. Proteases in mast cell granules are stored in their active forms [39]. The functions of mast cell proteases in inflammation and tissue repair [40], and those of protease-proteoglycan complexes in innate immunity [41] have been reviewed. These proteases are well recognised as pharmacological targets [42].

Mast cell protease storage is dependent on serglycin, and is moreover dependent on the presence of heparin and/or chondroitin, as demonstrated in NDST2^−/−^ mice that do not make the fully sulphated heparin structure [43, 44] and GalNAc4S-6OST^−/−^ mice that cannot generate the CS-E structure [45]. No evidence has been reported that fully anticoagulant heparin, containing the 3-OST product sequence, is necessary for protease storage. Mouse CTMCs deficient in a combination of proteases have much reduced heparin, but unaltered chondroitin content [46]. Protease deficient, serglycin deficient, and heparin deficient mast cells all display altered granule morphology [43, 44, 46, 47].

**Fig. 4:**
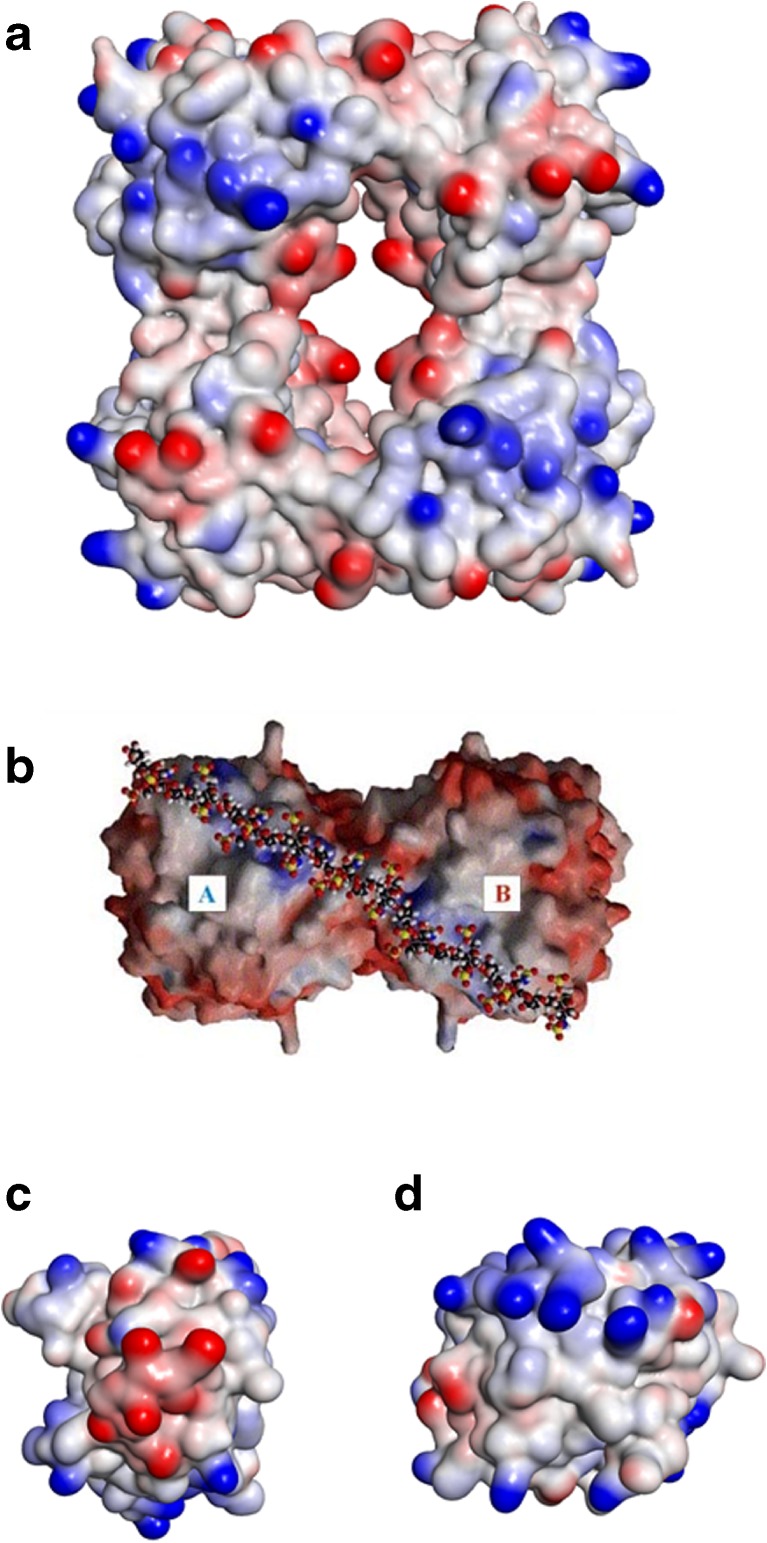
Heparin interactions with proteases **a** A β-tryptase tetramer crystal structure (1A0L.pdb), coloured by charge where blue is positive and red negative. This view clearly shows the restricted central active site. Clusters of basic residues, coloured blue, might accommodate a heparin chain binding to two monomers and so stabilising the active tetramer. Diagram made in Discovery Studio Visualiser, Accelrys Software Inc. **b** An α-tryptase tetramer crystal structure (1LTO.pdb), turned over by 90° to show the equivalent of the top face as compared with A). This view shows an almost continuous line of basic amino acid residues aligning with a long heparin molecule, shown in ball and stick format. Reproduced from [50] with permission. **c** A chymase monomer crystal structure (4KP0.pdb), showing clusters of basic amino acid residues (blue) on opposite sides of the charge-coloured surface, with a cluster of acidic residues (red) between them. The orientation of these two basic patches on the surface of chymase might allow this enzyme to pack between heparin chains of serglycin. The active site groove is on the left face of this diagram. Diagram made in Discovery Studio Visualiser, Accelrys Software Inc. **d** A granzyme B monomer crystal structure (1IAU.pdb) showing a substantial cluster of basic residues (blue) at the top of the charge-coloured surface. The active site is on the left face of the diagram. Diagram made in Discovery Studio Visualiser, Accelrys Software Inc

In the mast cell granule, proteases and heparin are in intimate contact, so extensive heparin binding sites containing basic residues are expected on the surfaces of the proteases. For tryptases, dependent on heparin for activity and stabilisation of the tetrameric form [48], a plausible cross-linking geometry has been described for human β-II tryptase [49] (Fig. 4a) and a potentially heparin-binding linear area of basic character has been identified across two monomeric units in the tetramer of human α1-tryptase (Fig. 4b) [50]. Besides the active tetrameric form, stabilised by heparin, tryptases such as human β-tryptase and mouse mMCP-6 can also form active monomers in the presence of heparin, even if the heparin chain is too small to bridge two protein monomers (though heparin of such a low molecular weight is not likely to be found *in vivo*) [51, 52]. The minimum length of heparin for complex formation with tryptase tetramer is 20 monosaccharides [53]. The substrate selectivities for monomeric and tetrameric tryptases are different, and the monomeric form is susceptible to inhibition by protein inhibitors such as BPTI [51]; in the tetrameric form the active sites of the four monomers face each other round a restricted space in the centre of the complex, restricting access to inhibitors that are proteins.

As the tetramer is most stable at low pH and in the presence of heparin, it has been proposed that tryptases are stored in the tetrameric form [54], and the presence of two separate, linear heparin binding sites on each tetramer, requiring long heparin chains, is compatible with the formation of closely packed complexes with heparin chains attached to a serglycin core. Depolymerisation of heparin, degranulation, and rise in pH could dissociate the tetramers to give heparin-activated, effective monomers, or inactive monomers as the heparin dissociates over time.

It is interesting to note that antithrombin is capable of inhibiting the monomeric active form of tryptase [55]; so heparin can either activate the serine protease or potentiate its inhibition if all three are found together. Tetrameric, heparin-associated human tryptase can prevent coagulation and formation of fibrin deposits in inflamed tissue by cleaving fibrinogen [56].

For human chymase and granzyme B, typical heparin/HS binding sites can be identified. Chymase is active as a monomer, and does not require heparin for its activity. It has two separate well-defined patches of basic residues that may constitute heparin-binding sites, on opposite faces of the protein surface (Fig. 4c). There is only one human chymase (rodents have several), and its presence or absence determines the classification of human mast cells into MC_TC_ mast cells, positive for both tryptase and chymase, and MC_T_ cells, positive for tryptase only [2]. These two cell types are considered approximately equivalent to rodent CTMCs and MMCs, respectively. It is likely that this classification is an oversimplification of the true situation, at least in tissues like the lung [57]. Granzyme B has a particularly clear basic patch, shown on the protein surface in Fig. 4d. All of these potential heparin binding sites on the proteases have different sizes and shapes; they may have different affinities for heparin, different abilities to bind to more than one heparin chain in the granule, different requirements in terms of heparin chain length, and even possibly different preferences for fine structure within heparin. It is also possible, of course, that preferences for CS/DS structures vs heparin structures differ between proteases.

## Heparanase and the depolymerisation of serglycin

In 1971, Ogren and Lindahl [58] described enzymatic depolymerisation of mouse macromolecular heparin to yield heparin chains of about the range of molecular weights found in commercial samples.

The average molecular weight of heparin sodium USP is somewhere between 15 kDa and 19 kDa; not more than 20% material by weight is over 24 kDa, and about 10% is less than 8 kDa [59]. As far as it is possible to tell, the molecular weight distributions of commercial porcine mucosal heparin have fallen roughly within that range for decades [60]; the USP’s formal limits have only recently been introduced [59, 61]. Differences in manufacturing processes have only a minor influence over molecular weight [59], so for the most part, commercial heparin retains the molecular weight it had on extraction from hog mucosa.

The endoglucuronidase known as heparanase has been shown to depolymerise heparin in the mast cell granule [62] and is thought to play an important part in maintaining homeostasis in granules; overexpression of heparanase reduces heparin chain size and protease storage capacity, whereas lack of heparanase leads to increase in heparin chain length and protease content [63]. The preferred substrate sequence for heparanase is at the junction between non epimerized, less sulphated sequences and the non-reducing side of highly sulphated sequences, such as at the GlcA-GlcNS6S linkage [64]. Heparin, largely made up of IdoA2S-GlcNS,6S disaccharides, is therefore cleaved not into short oligosaccharides, but into longer lengths, giving rise to the molecular weight profile of commercially produced heparin. Mast cells vary in their heparanase content [63]; rat skin mast cells presumably contain little heparanase, so that complete serglycin, or macromolecular heparin, can be prepared from them in spite of the fact that it provides a substrate for heparanase [62, 65]. The partial depolymerisation of serglycin heparin by heparanase has been found to aid the release of proteases on degranulation [66]. No equivalent enzyme for the depolymerisation of mast cell granule CS/DS has been identified, as far as we know.

## Mast cell GAGs after degranulation

On mast cell degranulation, a substantial list of pre-formed mediators are released into the extracellular space, among them the biogenic amines histamine and serotonin, the mast cell specific proteases described above, other non-mast cell specific proteases, cytokines and of course serglycin proteoglycan and heparanase-cleaved heparin polysaccharide [39]. Presumably also the chondroitin/dermatan sidechains of serglycin are released. The effects of high concentrations of these mediators in a limited three-dimensional space, and the differential time courses of diffusion and breakdown of protease-serglycin complexes that eventually disperse these agents, is not yet completely understood. It has been found that mouse mast cell protease-7 (mMCP-7) disperses away from the site of degranulation into the blood more quickly than mMCP-6: this is consistent with the histidine-based heparin binding site of mMCP-7, which loses affinity at neutral pH, as compared with the lysine and arginine based heparin binding site of mMCP-6, which does not [67]. Heparin can have a stabilising and activating influence on protease activity, and can also potentiate serine protease inhibitors, so its role may change according to the relative concentrations of proteins and GAGs over time.

Heparin has been shown to be released extracellularly after IgE mediated degranulation of mast cells from human lung tissue [68] and has been proposed to act as a homeostatic “braking” mechanism limiting the extent of inflammation in an analogous way to homeostatic controlling mechanisms limiting the actions of neurotransmitters and hormones in the nervous and endocrine systems [69]. However, there are only limited data on the biological activity of endogenously released GAGs. Thus, heparin derived from rat mast cells has been shown to have anti-proliferative activity for vascular smooth muscle cells [70] and more recently we have demonstrated that endogenous heparin obtained from rat peritoneal mast cells inhibits the adhesion of neutrophils to vascular endothelial cells *in vivo* and the recruitment of inflammatory cells into the peritoneum [35]. Nonetheless multiple studies have now shown that heparin can bind to many proteins involved in different stages of the inflammatory cascade involved in the recruitment of inflammatory cells from blood into extravascular tissues, including adhesion to vascular endothelium, rolling on endothelial cells and the subsequent diapedesis across the endothelium [71]. This is as a consequence of many of the adhesion molecules involved in this cascade having heparin binding domains in their structure. Once bound, heparin inhibits the adhesion molecule recognising its counter ligand and thus prevents the physiological function that would normally have arisen if the adhesion molecules were allowed to bind. Furthermore, heparin can bind to certain chemokines that can trigger the recruitment of leukocytes into tissues [72], as well as binding to a number of cationic pro-inflammatory molecules released from activated leukocytes such as neutrophil elastase and eosinophil derived major basic protein [7]. By binding these cationic molecules heparin effectively neutralises their ability to cause tissue damage. Not surprisingly therefore exogenously administered heparin has been shown to have a wide variety of anti-inflammatory actions *in vivo*, both experimentally and clinically (reviewed in [7]).

## Heparin from different tissues and species

Currently, heparin for medicinal use in the USA and Europe is prepared from porcine mucosa, and in other parts of the world bovine mucosal heparin is in use. Heparins from these sources have been compared by spectroscopic, molecular weight determination and degradative analyses, and found to be different in their disaccharide compositions [6, 73–75]. In the past, bovine lung heparin was available, but fell out of use partly for cost reasons and partly due to caution in the use of bovine-derived products in the wake of the discovery of transmissible spongiform encephalopathies (TSEs) in cattle [76].

Some exploration of other species and tissues has taken place, for example the preparation of a raw heparin from dromedary intestine [77]; or a less promising alternative of heparin from an avian source, turkey intestine, that yielded only relatively low-sulphated, less active HS [78].

Heparin has been prepared for research purposes from many animal species, ranging from a classic study of tissue distribution of heparin in eight mammalian species including man to reports of heparin-like GAGs in marine invertebrates [79]. Molluscs, such as the mussel *Anodonta anodonta*, contain heparin similar to that from mammalian sources [80], and several species of clam contain highly anticoagulant heparin [81, 82]. A detailed analysis of such a heparin from the clam *Tapes phylippinarum* revealed that the pentasaccharide with high affinity for antithrombin has the same structure as that from bovine and porcine mucosal heparin [83] (Fig. 2c). Clam heparin occurs in mast-like cells near epithelial surfaces [84, 85]. Ascidian test cells containing heparin-like structures in granules [86, 87] may well be related to mast cells [88]; like mast cells, test cells are likely to be involved in defence against infection [89]. Heparin has also been found in haemocytes of the ascidian *Styela plicata* [90], and this cell type may also be related to mammalian mast cells [1]. The idea that heparin has a common role in both vertebrates and invertebrates has been explored [91].

In most of these studies, heparin was prepared by fractionation of heparin from GAG mixtures obtained from whole tissues, rather than from isolated mast cells. It is necessary to assume, in these cases, that HS-type glucosaminoglycans with a high degree of sulphation and substantial anticoagulant activity represent mast cell derived heparin, rather than heparan sulphate from other cell types. This assumption is not unreasonable for heparin but cannot at all be made for chondroitin sulphates; we have almost no information on the variability of mast cell chondroitins between species.

Particularly interesting is human heparin, prepared from a large hemangioma, that has been compared in detail with porcine mucosal heparin; heparins from the two sources are similar, with human heparin having, if anything, higher anticoagulant potency [92]. Heparin from human placenta has also been prepared [93].

It is also interesting to note the variability in heparin sequence and structure between different tissues in the same species. A comparative study of 12 tissues in eight species yielded heparins with a variety of different molecular weights and anticoagulant activities, all of which gave trisulphated disaccharide on digestion with heparinase I [94]. Commercial heparin derived from bovine lung and from bovine intestinal mucosa are clearly and consistently different in structure [75]. Mast cells in different environments do not simply choose between heparin and chondroitin GAGs, but mobilise the sets of biosynthetic enzymes that determine the fine structure of heparin and chondroitin in different ways.

## Conclusions

Though the distinction between CTMCs and MMCs in rodents has justification, it is probably not safe to assume that this simple dichotomy can be extended to all tissues and all species. Even if it is true that less heparin is present in MMCs than in CTMCs, enough heparin can be obtained from the intestinal mucosae of large food animals to support a substantial pharmaceutical industry. Mast cells have the apparatus to assemble any serglycin the local tissue environment requires.

Different heparin structures determined for different tissues within the same species indicate that mast cell populations differ not only in the proportions of different GAGs, but in the fine structure of those GAGs as well. However, there are strong similarities in heparin structures across the entire animal kingdom, including the presence of the pentasaccharide sequence with high affinity for the serpin antithrombin. Serpins are present in all multicellular eukaryotes [95], so are at least as ubiquitous as glycosaminoglycans, and they are not all, like antithrombin, concerned with blood coagulation but inhibit proteolytic cascades in a number of contexts [96]. The functional significance of mast cell GAG fine structure may be concerned with the role of heparin after degranulation, and may be involved in inflammatory or anti-pathogenic roles of mast cells [79, 93].

In summary, there remain many gaps in our understanding of the structure and function of mast cell GAGs. We know that their overall anionic nature is crucial to correct packing of granule proteases, but we have no functional explanation for the differences in GAG fine structure that exist between species and tissues. There is a great deal of scope for future work in this field.
